# Socio‐economic and dietary diversity characteristics are associated with anemia among pregnant women attending antenatal care services in public health centers of Kembata Tembaro Zone, Southern Ethiopia

**DOI:** 10.1002/fsn3.1485

**Published:** 2020-03-06

**Authors:** Semalign Samuel, Teshale Darebo, Derese Tamiru Desta, Afework Mulugeta

**Affiliations:** ^1^ TB Epidemiology Technical Advisor at Ministry of Health Addis Ababa Ethiopia; ^2^ Department of Public Health Mizan Tepi University Mizzan Ethiopia; ^3^ Schools of Nutrition, Food Science and Technology Hawassa University Hawassa Ethiopia; ^4^ School of Public Health Mekele University Mekele Ethiopia

**Keywords:** anemia, dietary diversity, hemoglobin, pregnant women

## Abstract

Anemia affects every country in the world including Ethiopia. It costs the socio‐economic developments due to its health impacts. This study was aimed to determine socio‐demographic and dietary diversity and other factors associated with anemia among pregnant women in Kembata Tembaro Zone. Institution‐based cross‐sectional study design was employed. Data were collected from 423 pregnant women. Hemoglobin level was measured by Hemocue. Pretested interviewer‐administered close‐ended questionnaire was used to collect the data. Analysis was done using SPSS for windows version 20. Descriptive and multivariate logistic regression analysis was conducted. We found that overall prevalence of anemia was 18%. Less than half (43.3%) of the respondents visited the health centers for antenatal care follow‐up at least once during their pregnancy time. Only 20.1% consumed more than five food preceding 24 hr. 59.3% of the respondents were taking iron supplements during data collection time. Anemia was significantly associated with the lowest wealth index, formal education, women's occupation, husband occupation, low dietary diversity, and inadequate intake of iron‐rich foods, history of malaria infection, maternal age, and parity. Anemia was of mild public health concern in the study area. The uptake of antenatal care service for the recommended number of visits and iron folic acid supplementation and the diet diversity was low in the pregnant women. The prevalence of anemia was mild public health concern. The early and timely uptake of iron folic acid supplementation was poor. Dietary diversity and consumption of iron‐rich foods was found to be low. Thus, community mobilization and health education to improve the uptake of the antenatal care services, increase micronutrient intake through food based and control of infections are recommended.

## Graphical abstract

Socio‐economic factors are associated with anemia. Dietary diversity score of pregnant mother is related with anemia.

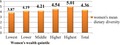

## 1 INTRODUCTION

Anemia affects every country in the world. Basically, it costs the socio‐economic developments due to its health impacts (Benoist, McLean, Egli, & Cogswell, 2008). There are various factors which contribute to the onset of anemia. From the major factors, low level of iron nutrient intake and infections like malaria play an important role for the occurrence of anemia. The main function of the iron as a nutrient is carrying oxygen, and its low level in the diet mainly which is manifested in the large proportion of humans across the globe is the main factor for it (Zimmermann, 2007), but also it often works in symphony with folate and/or vitamin B_12_ deficiency (Milman, 2014).

These micronutrient deficiency anemias, particularly of iron combined with infections such as malaria, are common in Ethiopia (Ezechi Oliver, 2012). Worldwide, it is estimated that about 20% of maternal deaths are caused by anemia; in addition, anemia contributes partly to 50% of all maternal deaths. Similar situation is found in sub‐Saharan Africa where anemia is reportedly accounted for about 20% of all maternal deaths (Oladunjoye, 2012). Anemia among the pregnant women has both maternal and fetal impacts (Milman, 2014). It causes increased risks of mortality among the pregnant women, neonatal low birthweight and death and perhaps it increases the probability of having a lower cognition among the infants and later in their life courses (USAID, 2011).

The 2016 Ethiopian Demographic and Health Survey (EDHS) reveals women have aged 15–49 for anemia. Nearly one‐fourth (24%) of reproductive‐aged women (15–49 years) in Ethiopia are anemic. Since 2005, anemia among women has slightly decreased from 27% to 24% in 2016. The prevalence was high in the rural area (25%) compared with the urban area (17%) (CSA, 2016). Additionally, 2011 EDHS indicates greater proportion of pregnant women were anemic (22%) compared with the mothers breastfeeding (19%), and women who are neither pregnant nor breastfeeding (15%). The prevalence also differs by residence areas (urban vs. rural). Greater proportion of women in rural areas were anemic (18%) compared with the urban areas (11%). From the other socio‐demographic characteristics, educational status was a factor for anemia. Thus, women with no formal education got double probability of having anemia compared with the women who attended secondary school and above school. Correspondingly, the prevalence was decreased as the wealth status of the household becomes better (CSA, 2011).

Irrespective of the national‐level study, careful examination of the health institution‐based reports exposed that anemia has been a serious health issue among the pregnant women in the study area. Moreover, the staple diets in the community are plant‐based foods known to reduce iron absorption. Malaria and intestinal parasites are also in the top leading causes of morbidity; however, the most contributing factor for anemia is not well known specifically in Kembata Tembaro Zone, Ethiopia. Thus, this study is aimed to assess the prevalence of anemia and factors associated with anemia among pregnant women attending antenatal care service in public health centers.

## 2 MATERIALS AND METHODS

### 2.1 Study area

The study was carried out from August to September 2014 in public health centers of Kembata Tembaro Zone, Southern Ethiopia. The zone is one of the 15 zones in Southern Nations and Nationalities People Region (SNNPR). The capital town of the zone, Durame, is located 306 km from Addis Ababa through Hosanna, in the southwest direction. The town has altitude about 2,100 m above sea level (masl). The zone is divided into seven districts, one town administration, 120 rural kebeles and 22 urban kebeles/localities, and 141,952 hectares of land. Kembata Tembaro Zone with a total population of 869,686. Women in the reproductive age constitute 23.3% of the population (196,457) and estimated pregnancy is 3.6% of total population, which gives us 30,354. It is geographically bordered by Dawuro zone (west), Wolayita zone (south), Hadiya zone (north), and Alaba special district and Hadiya zone (east).

At the time of data collection, the zone contains one general hospital, 28 governmental and 3 non‐governmental (NGO) health centers, 135 health posts, and more than 15 private clinics serving the community (Kembata Tembaro Zone Health Department, 2013).

### 2.2 Study design and population

The study implemented institution‐based cross‐sectional study design. The study participants were pregnant women who were selected during their visit in the selected public health centers in the study area for antenatal care. Those pregnant women who fulfill the inclusion criteria were enrolled in the study regardless of their number of visits. Each participants were recruited to the study only once on their all visits during the study period.

### 2.3 Sample size and sampling technique

A total of 436 pregnant women were selected for the study. Single population proportion formula shown below was used with the assumption of 95% confidence level (1.96), margin of error of (0.05), prevalence of (22%) (CSA, 2011), and design effect of 1.5.

(1) $$n=\frac{{\left(Z\alpha /2\right)}^{2}P\left(1-P\right)}{d^{2}}$$

where *n* = required sample size. *Zα*/_2_ = critical value for normal distribution at 95% confidence level which equals to 1.96. *d* = an absolute precision (margin of error 5%).

Regarding sampling technique, of the 15 zones and four special district of SNNPR, Kembata Tembaro Zone was selected purposively. The zone contains 28 public health centers; ten of the health centers were selected randomly. Then, the study units were assigned for each health center using proportional allocation method based on their catchment population. Lastly, systematic sampling method was used to include pregnant mothers who fulfill the inclusion criteria. The list of pregnant women found at each health center and the last three‐month average report review were used to estimate the number of pregnant women that would visit the health centers for antenatal care (ANC). The first respondent was selected by lottery method, and K = *N*/*n* (where *N*, average 1‐month report and *n* = allocated sample size for each health center) interval was used to select the next respondent.

### 2.4 Variables

Anemia was considered as dependent variable. The independent variables were socio‐demographic variables and economic factors, maternal‐related factors, and health‐ and nutrition‐related characteristics.

### 2.5 Data collection procedures

Data were collected during August 8 to September 22, 2014. Questionnaires were prepared and pretested. The main contents of the questions were socio‐demographic characteristics, wealth index, obstetric and gynecological history (trimester, gravidity, parity, ANC follow‐up, iron supplementation, and others), and nutrition‐ and health‐related variables. A tool about dietary intake (FAO, 2011) was developed and adapted to the local context. A predefined 9 food group with 24‐hr dietary reference was applied to assess the dietary intakes. The food groups were (starchy staples (cereals, roots, and tubers); legumes, nuts, and seeds; milk and milk products; organ meat; meat and fish; dark green leafy vegetables; other vitamin A‐rich fruits and vegetables; other fruits and vegetables, and eggs).

### 2.6 Blood sample collection and laboratory analysis

Capillary blood samples were used to assess hemoglobin concentration using HemoCue^®^ portable system, and the Hgb concentration was determined instantly after sample collection. Altitude was measured at the health centers using Magilan^®^ Global Positioning System (GPS). The hemoglobin concentration was adjusted for altitude according to WHO recommendation (WHO, 2011a, 2011b).

### 2.7 Quality control

Data were collected by trained diploma and bachelor degree nurses. One data collector for each health facility and four supervisors were recruited to overlook the entire data collection. In addition, one laboratory technician for each facility was recruited for blood sample collection and hemoglobin determination. The interview tools were adapted according to the existing scientific literatures and contextualized as per the locality and objectives of the current research. The interview tools were first prepared in English language and translated to the local language (Kembatigna) and back‐translated to English by the native speakers of the languages. Later, two‐day training was given for data interviewer and supervisors on the data collection tools. The consistency of the data was checked by the principal investigator and supervisors in daily basis. The accuracy of HemoCue^®^ had been checked daily by the use of control cuvettes provided with the machines.

### 2.8 Data analysis

The data were entered and cleaned using Epi_info software version 3.5.1, and the analysis was done using statistical package for social sciences (SPSS) for windows, version 20. Frequencies, percentage, mean, and standard deviation were used for the descriptive analysis. Bivariate analysis was done to see the association of each independent variable with the outcome variable. Those variables having *p*‐value <.2 were entered into the multivariable logistic regression model to identify the effect of each independent variable with the outcome variables. A *p*‐value of <.05 was considered statistically significant, and adjusted odds ratio with 95% CI was calculated to determine association. Variables were entered into the model using the “backward” method. During the analysis, the fitness and statistical assumptions of the logistic model, Hosmer and Lemeshow statistics, and model summary table were checked.

Principal component analysis (PCA) was conducted to construct the wealth index. The main variables included in the analysis were related to ownership of selected household assets, size of agricultural land, quantity of livestock, materials used for housing construction, and ownership of improved sanitation facilities. Lastly, the scores were divided into five equal wealth quintiles (lowest, lower, middle, higher, and highest).

The dietary diversity score (DDS) was computed from the nine food groups. Consuming a food item from any of the aforementioned groups earned a score of 1 for that specific category. If not, a score of 0 was given. Accordingly, a score of nine score points was developed. Later, the scores were categorized into three categories: low (scores ≤3), medium (scores = 4 or 5), and high (scores ≥6) groups (FAO, 2011).

## 3 RESULTS

### 3.1 Socio‐demographic characteristics of the respondents

Of the 436 respondents recruited, 423 (97%) volunteered to participate in the study. The mean age of the respondents was 26.85 with standard deviations of ±5.3 years, while their ages ranged from 18 to 43 years. Of the respondents, 256 (60.5%) were from rural areas and the rest 167 (39.5%) were urban dwellers. Almost all (98.8%) of the study participants were married at the time of data collection (Table 1).

**Table 1:** Distribution of pregnant mothers visiting health centers for the ANC follow‐up by their socio‐demographic characteristics, Kembata Tembaro Zone, Sothern Ethiopia, 2014 (*n* = 423)

Characteristics	No.	%
Marital status		
Married	418	98.8
Single	5	1.2
Age in years		
15–24	134	31.7
25–34	240	56.7
35–44	49	11.6
Residence		
Rural	256	60.5
Urban	167	39.5
Educational level		
No formal education	130	30.7
Primary (1 – 8)	190	44.9
Secondary (9 – 12)	76	18
Above secondary	27	6.4
Family size		
1–5	273	64.5
>5	150	35.5
Wealth Index		
Lowest	85	20.1
Lower	88	20.8
Middle	87	20.6
Higher	79	18.7
Highest	84	19.9

### 3.2 Prevalence of anemia from the laboratory findings among the pregnant women

**Figure 1:**
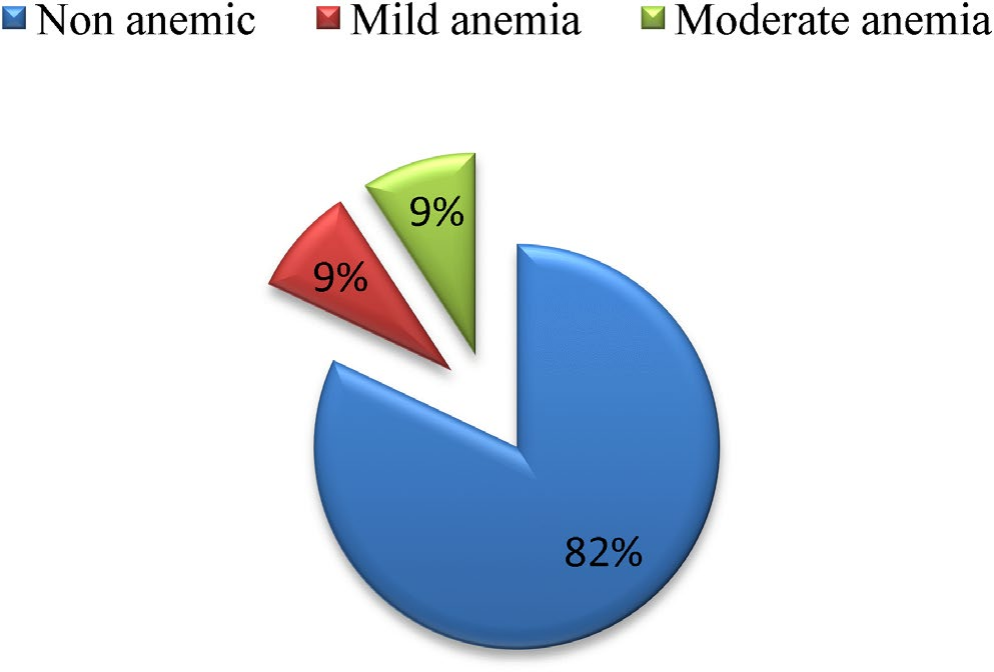
Anemia classification of pregnant women attending health centers for their ANC follow‐up in Kembata Tembaro Zone, Southern Ethiopia, 2014 (*n* = 423)

Prevalence of anemia (Hgb <11 g/dl, adjusted for altitude) among the pregnant women was found to be 18%. Meanwhile, the mean (*SD*) Hgb level was 12.26 (±0.07) g/dl (95% CI: 12.13–12.41 g/dl). The level for each trimesters was as follows 12.85 (±1.43) g/dl (first trimester), 12.35 (±1.6) g/dl (second trimester), and 12.27 (±1.6) g/dl (third trimester). Again, the prevalence for the women who do not know their gestational age was 11.7(±1.4) g/dl. The occurrence of mild and moderate anemia was 37 (8.8%) and 39 (9.2%), respectively (Figure 1). Besides the laboratory finding of hemoglobin level to diagnose anemia was supported by clinical findings (history and physical examination). Of the total 45 pale cases, 35 (77.8%) had hemoglobin concentration <11.0 g/dl. Those with previous history of anemia and those who had at least one symptom of anemia during their current pregnancy became anemic than their counterparts.

### 3.3 Obstetric characteristics among the pregnant women

Majority, 260 (61.5%) of the respondents were in their third trimester, and 101 (23.9%) of them were in their second trimester, and 24(5.7%) were in their first trimester. The rest did not know their gestational age. About 43.3% of the respondents visited the health centers for antenatal care for the first time, 115 (27.2%) women visited for second time, 69 (16.3%) visited for third time, and 56(13.2%) visited for four and above times during the current pregnancy. More than half, 251(59.3%) of the respondents were being supplemented iron during the time of the interview and 172 (40.7%) did not receive. The study participants were also asked if there were any local and cultural barriers for IFA intake and only three women said and believe IFA tablets make the fetus bigger for gestational age.

### 3.4 Dietary diversity and other related characteristics of the pregnant women

Of the nine food groups, starchy staples (cereals, roots, and tubers) were consumed by all women, dark green leafy vegetables by 69.7%, legumes, nuts, and seeds by 66.9%, milk and milk products by 66.4%, other fruits and vegetables by 57.7%, meat and fish by 31.4%, vitamin A‐rich fruits and vegetables by 20.3%, eggs by 17.7%, and organ meat by 5.7%. Approximately, a quarter (28.1%) of the women consumed less than three food groups, 219 (51.8%) consumed 4–5 food groups and the rest 85 (20.1%) consumed more than five food groups during the last 24 hr. One hundred fifty‐seven (37%) women consumed iron‐rich foods (meat and fish + organ meat) during the 24 hr prior to the data collection. Majority, 367 (86.8%) of women took coffee and/or tea within an hour after meal, while in contrary only 58 (13.7%) of pregnant women took fruits or fruit juice after meal. Women infected by malaria and intestinal parasites during the current pregnancy were 24 (5.7%) and 55 (13%), respectively. Women, who at least heard about anemia, have previous history of anemia, had at least one sign/symptom during their current pregnancy, who know at least one cause and preventive methods of anemia, and who know at least one sign/symptom of anemia were 277 (65.5%), 29 (6.9%), 66 (15.6%), 248 (58.6%), and 252 (59.6%). respectively (Table 2).

**Table 2:** Distribution of pregnant mothers visiting health centers for the ANC follow‐up by their obstetric, nutritional, and medical characteristics, Kembata Tembaro Zone, Southern Ethiopia, 2014 (*n* = 423)

Characteristics	No	%
Number of alive children		
0	135	31.9
1–2	157	37.1
3–4	96	22.7
>4	35	8.3
Birth interval (*n* = 293)		
<2 years	36	12.3
>/=2 years	257	87.7
Place of birth for last baby (*n* = 293)		
Health Institution	161	54.9
Home	132	45.1
Gestational age		
1st trimester	24	5.7
2nd trimester	101	23.9
3rd trimester	260	61.5
Do not know	38	9
Taken IFA		
No	172	40.7
Yes	251	59.3
WDDS		
Lower	119	28.1
Middle	219	51.8
Higher	85	20.1

### 3.5 Socio‐demographic and other factors associated with anemia

In the current study, it is revealed that wealth index, respondents educational status, women's occupation, husband's occupation, age of the women, and their residence variables were significantly associated with occurrence of anemia. Respondents from the lowest wealth quintiles had higher risk of developing anemia in contrast to highest wealth quintiles (AOR = 5.03, 95% CI = 1.07–23.7). Pregnant women with no formal education were the most affected compared with their counterparts (AOR = 6.26, 95%CI = 3.11–12.57). Daily laborers were more affected (AOR = 3.17, 95%CI = 1.1–9.14) than housewives. The pregnant women who were daily laborers were also highly affected by anemia (AOR = 3.1, 95%CI = 1.25–7.64) compared to farmer occupation. Moreover, the pregnant women residing in the rural did get highest odds of having anemia (AOR = 2.43, 95%CI = 1.08–5.44 (Table 3).

**Table 3:** Selected socio‐demographic, health and dietary factors associated with anemia among pregnant women attending public health centers for their ANC follow‐up, Kembata Tembaro Zone, SNNPR Ethiopia, 2014 (*n* = 423)

Variables	Hgb in g/dl mean (±sd)	Anemia		COR, 95% (CI)	AOR,95% (CI)
		Yes	No		
Iron‐rich food taken?					
No	12.03 ± 1.61	220	127	4.91 (2.29–10.54)	4.3 (1.75–10.53)
Yes	12.75 ± 1.38	68	8		1
Wealth index					
Lowest	11.72 ± 1.68	29	56	10.36 (3.45–31.12)	5.03(1.07–23.7)
Lower	12.28 ± 1.9	33	65	7.08 (2.33–21.5)	3.33(0.78–14.2)
Middle	12.2 ± 1.56	15	72	4.17 (1.32–13.13)	1.56 (0.36–6.6)
Higher	12.32 ± 1.08	5	74	1.35 (0.35–5.22)	0.59 (0.11–3.01)
Highest	12.82 ± 1.3	4	80		1
Residence					
Rural	12 ± 1.57	59	197	2.64 (1.48–4.72)	2.43(1.08– 5.44)
Urban	12.7 ± 1.5	17	150		1
Parity					
No	12.64 ± 1.5	14	116		1
Yes	12.1 ± 1.58	62	231	2.22 (1.2–4.14	3.9 (1.57–9.7)
Formal education					
No	11.4 ± 1.63	51	79	6.92 (4.03–11.88)	6.26(3.1–12.57)
Yes	12.66 ± 1.4	25	268		1
WDDS					
Lower	11.62 ± 1.58	39	80	3.66 (1.71–7.84)	‐
Middle	12.41 ± 1.4	27	192	1.06 (0.49–2.28)	‐
Higher	12.78 ± 1.7	10	75	1	‐
IFA supplementation					
No	12.11 ± 1.66	38	134	1.59 (0.97–2.62)	1.7 (0.9–3.24)
Yes	12.37 ± 1.5	38	213		1
Number of ANC visits					
First	12.06 ± 1.68	41	142	1.32 (0.62–2.86)	
Second	12.51 ± 1.41	12	103	0.53 (0.23–1.33)	
Third	12.37 ± 1.45	13	56	1.07 (0.43–2.66)	
Fourth and above	12.31 ± 1.6	10	46	1	

**Figure 2:**
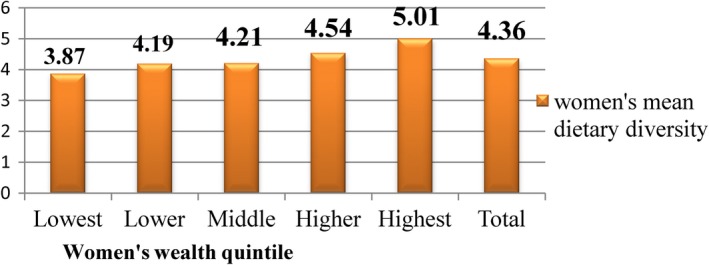
Mean WDDS of pregnant women attending health centers for their ANC follow‐up by their wealth index in Kembata Tembaro Zone, Southern Ethiopia, 2014 (*n* = 423)

**Figure 3:**
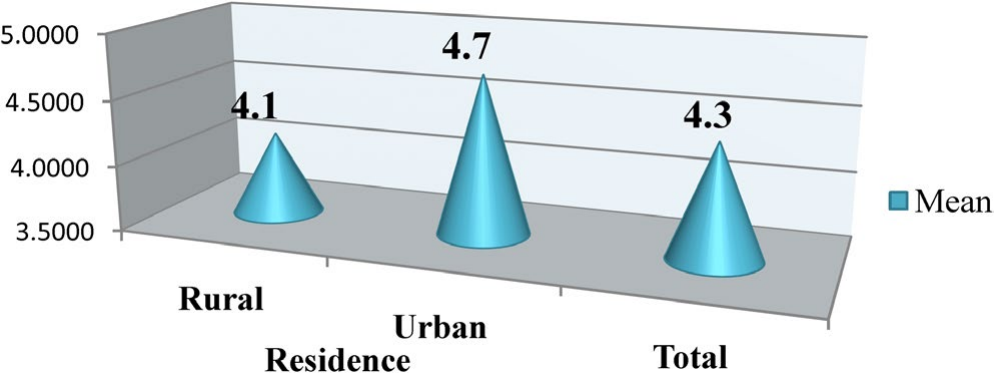
Mean WDDS of pregnant women attending health centers for their ANC follow‐up by their residence in Kembata Tembaro Zone, Southern Ethiopia, 2014 (*n* = 423)

Beyond socio‐demographic factors, dietary, obstetric, and other healthcare‐related correlates were also associated with anemia. The likelihood of having anemia among the respondents with lowest dietary diversity (DDS ≤3) was found to be 3.66 times with 95%CI = 1.71–7.84 compared with higher dietary score (DDS ≥6. Similarly, respondents who does not eat iron‐rich foods in the previous day of data collection had the odds of 4.3, 95% CI = 1.75–10.53). Dietary diversity was found to be better with the highest wealth quintiles of socio‐economic status, urban residence, and higher educational level (Figures 2 and 3).

History of malaria attack was significantly associated with increased anemia (AOR = 7.86, 95% CI = 2.67–23.11). Pregnant women who gave birth at least once were more affected than pregnant women with the first pregnancy (AOR = 3.9, 95%CI = 1.57–9.69). In contrast to pregnant who received iron folic acid tablets, the odds of anemia was (AOR = 1.7, 95%CI = 0.91–3.24) higher elevated than women who did not receive the tablets, though the association was not significant (Table 3). The hemoglobin levels and prevalence of anemia were not statistically different across variables of gestational age, number of antenatal care visits, iron folic acid intake, frequency of coffee intake, place of birth, and birth interval in multiple variable logistic regressions.

## 4 DISCUSSION

The prevalence of anemia among pregnant women in the present study was 18%, 95%CI = 14.4–21.1 indicating that anemia in pregnant women was of mild public heath significance in the study area as per the World Health Organization (Benoist et al., 2008). The prevalence was closer but lower than the national prevalence (22% in 2011 and 24% in 2016) but higher than the regional prevalence (11%) reported in the EDHS (2011) (CSA, 2011, 2016). It is also comparable to the findings from Addis Ababa (21.3%) (Hailu, 2014) and Gonder (21.6%) (Alem et al., 2013) but it is higher than the findings reported from Debre Berhan (9.7%) (Ayenew, Abere, & Timerga, 2014). The possible reason for the difference might be the methodology used to collect the data. Unlike the current study, the Debre Berhan's study used a convenient method to include respondents into the study, lower sample size, and the hemoglobin findings were not adjusted for altitude. The finding of this study was however very low compared to a report from India, 94.3% (Kendre, 2014). It is suggested that the variations may be attributed to the different causes of anemia and dietary differences, population differences, and difference in methodology used in determining hemoglobin levels (Sahlis method vs. Hemocue method).

In the current study, 8.8% were of mild type of anemia (Hgb 10.0–10.9 g/d1), and 9.2% were moderate anemia (7–9.9 g/dl) cases. This finding correlates with the study done in Gonder, in which mild anemia 10.7%, moderate anemia (9.9%), and severe anemia (1%) were reported (Alem et al., 2013). The wealth quintiles of the respondents were significantly related with the occurrence of anemia. Pregnant women from the lowest wealth quintiles were five times more in a higher risk of having anemia, in contrast to the highest wealth quintiles. The current result is reliable and prove with other evidences from other developing and middle‐income nations (Balarajan, Ramakrishnan, Özaltin, Shankar, & Subramanian, 2011). For instance, finding from India reveals pregnant women from poorer socio‐economic quintiles were at increased risk of developing anemia (Vivek, Vivek, Halki, Maled, & Deshpande, 2012). Probably, the reason could be that women in the poorer households could have less access to education and financial freedoms. Thus, the financial constraints combined with wealth factors could cause inability for health services leading deleterious effects of infections like malaria.

In the current study, educational status was the other factors related with prevalence of anemia. Respondents with no formal education were affected 6.3 times than the educated women. This finding correlates with a finding the 2005 Demographic Health Survey (DHS) survey analysis. The analysis shows women with no formal education and who had primary level education had significantly higher risk of anemia with AOR of 2.59 (95% CI: 1.62–4.14) and 1.83 (95% CI: 1.13–2.96), respectively, than secondary and above level of education (Gebremedhin, 2014). A study in India also shows prevalence of anemia decreases with increased educational level of women. The prevalence of anemia was as high as 88.2% among illiterates while it was only 34.1% in those educated up to intermediate level (Swarnlatha, 2013). In this regard, educational level may improve awareness for good nutrition practices during pregnancy and during their course of life. Thus, the improved nutrition reduces the vulnerability of anemia among the pregnant women.

Maternal and husband occupations were related with increased risk of anemia in the current study. Odds of being anemic increases by 3.2 among daily laborers compared with housewife. Wives of daily laborers were affected three times, compared to wives of farmers. This finding is consistent with finding in Nepal, where severe anemia was significantly more prevalent among women worked for wages (Zeina Makhoul, 2012). It is obvious that the occupation of individuals is affected by their educational level and the place where they live. Most of the time those who are engaged in different daily labor and small business activities are poorly educated and the most economically disadvantaged categories, which in turn affect their practice toward healthy and nutritionally adequate foods. Foods rich in iron like beef, chicken, and fish are very expensive that those low income, the daily wage laborers cannot purchase them. Moreover, they miss their meals since they are extremely busy throughout the day.

The occurrence of anemia among the pregnant women residing in the rural was higher than in the urban area. Rural residents were 2.4 times more affected by anemia than the urban dwellers. Similar result was reported in Ethiopia with AOR of 1.99 (95% CI: 1.73–2.30) (Tadege, 2009). In another study from southeast Ethiopia, residence in rural area affected (AOR = 3.3, 95% CI: 1.5–7.4) compared to those in urban area (Kefiyalew, Zemene, Asres, & Gedefaw, 2014). The higher prevalence of anemia among pregnant women from rural areas is likely related to lack of information about adequate nutrition during pregnancy, economic factors, and inaccessibility of healthcare centers. Anemia was significantly associated with history of malaria attack, and the association was retained even when the variable was adjusted for its confounding effect in the logistic regression models. Similar findings were observed in a study done in Benin, 15% (95% CI = 13–17) of anemia in this population was attributable to malaria infestation (Oue´Draogo, 2012). A study among pregnant women in Gilgel Gibe dam area, Southwest Ethiopia also found that the odds of anemia was higher among pregnant women who were malaria infected (AOR = 11.19, 95% C.I: 3.31–7.7 (Getachew, Yewhalaw, Tafess, Getachew, & Zeynudin, 2012). It is a known fact that malaria is one of the factors which cause and aggravate the occurrence of anemia in pregnancy. This is because of hemolysis or breakdown of red blood cells, affected by malaria parasites which boost the increased demand for folates in pregnancy.

Dietary pattern of the pregnant women was one of the associated factors with increased chances of anemia. The respondents who had lower dietary diversity score were affected (COR = 3.66, 95%CI = 1.71–7.84) compared to higher scores. Those who did not eat iron‐rich foods within the last 24 hr have had odds of more than four times than their counterparts. This is comparable with evidence from Sidama, Southern Ethiopia, indicating the odds of anemia was 1.94 (95% CI: 1.15–3.27) times elevated in the low dietary diversity score category (Gebremedhin, 2014). Based on various food groups, cereal consumption was highest at 100 percent followed by consumption of dark green leafy vegetables (69.7%), legumes, nuts, and seeds (66.9%) but consumption of the most iron‐rich food groups such as meat and fish and organ meat was (31.4%) and (5.7%), respectively. Pregnancy is one of the critical times in which nutritional requirements increase. Hence, the pregnant women are expected to have higher dietary diversity score to meet the nutrient demands but in the current study, it was shown that the pregnant women were consuming less than the expected diversity scores.

In the present study, the main factors influencing dietary diversity were wealth index, their residence and education. Comparable findings are reported by other authors showing these aforementioned variables are the key related with dietary diversity Kenya (Kemunto, 2013). It is depicted that dietary diversity scores are nutrient adequacy among individual and household level (FAO, 2011). As the fetus continues to mature, there is an increase in nutritional needs in terms of macronutrients and micronutrients, and therefore, the pregnant woman is expected to increase their dietary intake in order to meet the needs. The chance of having anemia was observed rising with parity. Pregnant women who gave birth at least once were four times more affected than pregnant women with the first pregnancy. This finding correlates with the findings from the Jordanian study where a statistically significant increase (*p* < .01) was found between the overall prevalence of anemia in multipara women (64.0%) compared to primigravida women (49.3%) (Mohammad & Salahat, 2012). A previous study witnessed that the hemoglobin concentration decreases in grand multiparas compared with nulliparas (Gebremedhin, 2014). This shows that as the number of pregnancy increases, there might be depletion in nutrients thus the chances of having anemia increases.

Regarding iron folic acid intake, 59.3% of the pregnant women were supplemented during the current pregnancy; which is below the World Health Organization recommendation. This was highly attributed to lower and not timely visit of antenatal care visits. Majority, 164 (38.8%) of the pregnant women did not take iron folic acid because they were in their first visit during the study period. Of the 172 women who did not receive iron folic acid, only 22 were in their first trimester and the rest 150 were in their second trimester and above. About 97% of the pregnant women who visited the health centers previously were iron folic acid supplemented. The variables such as gestational age, antenatal care visit, birth interval, and family size were not statistically significant; however, many other studies demonstrated their association with anemia (Haidar, 2010; Hailu, 2014; Hoque, Kader, & Hoque, 2007; Kefiyalew et al., 2014).

### 4.1 Strength and Weakness of the study

Even if the current study had adequate sample and better response rate, there might be biases as it is institution‐based study which may exclude most at‐risk women. Hence, the occurrence of biases might have underestimated or overestimated the prevalence rates of anemia as well as the dietary intake of the women.

## 5 CONCLUSIONS

The prevalence of anemia was mild public health concern in the current study based on the World Health Organization standard. The early and timely uptake of the cheaper, faster, better, and evidence‐based focused antenatal care approach and iron folic acid supplementation was poor in the study area. Similarly, diet diversity which is considered to be the proxy indicator for micronutrient intake and consumption of iron‐rich foods was found to be low among the pregnant women. Being from the lower wealth index and educational status categories were also main affecting factors to anemia. Thus, the burden of anemia should be alleviated through improved uptake of the antenatal care services available in the health centers. Economic and educational empowerments like livelihood promotion strategies have affirmative inputs. Diet diversity and intake iron‐rich foods must be enhanced especially during pregnancy with effective nutrition education. To identify additional contributing factors for anemia, we recommend community‐based study using additional variables like weighed food scores so as to precisely determine the dietary intakes and its impacts on anemia.

## CONFLICT OF INTERESTS

All authors declare that they have no competing interests. The lead author affirms that this manuscript is accurate and transparent.

## ETHICAL APPROVAL

The study conforms to the Declaration of Helsinki guidelines for human subjects and approval was given by Hawassa University Institutional Review Board (IRB). Prior to data collection, the district administrators were contacted with a description of the study and purposes and consent was taken. The nature of the study was fully explained to the respondent and oral consent was taken ahead of interview. The respondents who were anemic and who had not started treatment were provided iron tablets and counseled on recommended nutritional options.
